# “A combined potential biomarker candidate and drug target quantified using Single Molecule Array ‐ GFAP and NFL implication in the progression of Dementia.”

**DOI:** 10.1002/alz.092680

**Published:** 2025-01-09

**Authors:** Masroor Anwar, Sharmistha Dey, Aparajit Ballav Dey, Jinkook Lee

**Affiliations:** ^1^ All India Institute of Medical Sciences, New Delhi, New Delhi India; ^2^ All India Institute of Medical Sciences, AIIMS, New Delhi, Delhi India; ^3^ Venu Geriatric Institute, South Delhi, Delhi India; ^4^ University of Southern California, Los Angeles, CA USA

## Abstract

**Background:**

The lack of knowledge about the onset and progression of dementia hampers its early diagnosis and possible treatment leading to severe condition like Alzheimer’s disease. Technological advancement like new brain network modeling tools might provide insight into the disease progression but a broader view of the biochemical changes induced by dementia in an early and pre‐symptomatic stage needs to be explored. Therefore, a multicentric longitudinal study was designed to quantify proteins molecules associated with cognitive impairment.

**Method:**

We measured the levels of four protein molecules Aβ1‐42, Aβ1‐40, GFAP, and NfL in plasma of 1619 participants comprising of 14% (Alzheimer’s Disease) AD patients, 39% (Mild cognitive impairment) MCI patients and 47% Geriatric controls (GC). The quantitative measurement was performed using flexible, robust, and ultra‐sensitive immunoassay platform SIMOA (Single Molecule Array). Statistical significance was defined as p < 0.05. All statistical analyses were performed in the Prism ver. 5.03 program (GraphPad Software Inc, La Jolla, CA, USA).

**Result:**

The comparative analysis showed plasma protein concentrations of NFL and GFAP were significantly higher in AD and MCI patients compared to GC group (p < 0.001) whereas no significant difference was observed for Aβ1‐42 and Aβ1‐40. Interestingly gender wise analysis of NFL showed the concentration difference between GC versus AD was significantly higher in female compared to male (concentration difference Male; 23.78pg/mL Female; 3.47pg/mL). NFL and GFAP exhibited higher correlation with increasing age. Concentration of GFAP significantly increased in all the three age groups (60‐70years p<0.01, 71‐80years p<0.001, 81years and above p<0.0001) in AD patients compared to GC.

**Conclusion:**

We showed a correlation between a profile of four plasma protein biomarkers in development and progression of dementia patients. Our findings indicate that higher plasma NFL and GFAP expression may be a biomarker for AD risk and could predict progression from prodromal to probable AD dementia.

